# Wearable electrochemical device based on butterfly-like paper-based microfluidics for pH and Na^+^ monitoring in sweat

**DOI:** 10.1007/s00604-024-06564-1

**Published:** 2024-09-07

**Authors:** Luca Fiore, Vincenzo Mazzaracchio, Arianna Antinucci, Roberto Ferrara, Tommaso Sciarra, Florigio Lista, Amy Q. Shen, Fabiana Arduini

**Affiliations:** 1 Department of Chemical Science and Technologies, University of Rome Tor Vergata, Via Della Ricerca Scientifica 1, 00133 Rome, Italy; 2 SENSE4MED, Via Bitonto 139, 00133 Rome, Italy; 3 Physical Medicine and Rehabilitation Unit, Italian Army Medical Hospital, 00184 Rome, Italy; 4 Defence Institute for Biomedical Sciences, Rome, Italy; 5 Micro/Bio/Nanofluidics Unit, Okinawa Institute of Science and Technology Graduate University, 1919-1 Tancha, Onna-Son, Okinawa, 904-0495 Japan

**Keywords:** Paper-based microfluidics, Wearable potentiometric sensor, Sodium and pH detection, Sweat analysis, Dehydration and energetic effort monitoring

## Abstract

A wearable potentiometric device is reported based on an innovative butterfly-like paper-based microfluidic system, allowing for continuous monitoring of pH and Na^+^ levels in sweat during physical activity. Specifically, the use of the butterfly-like configuration avoids evaporation phenomena and memory effects, enabling precise and timely biomarker determination in sweat. Two ad hoc modified screen-printed electrodes were embedded in the butterfly-like paper-based microfluidics, and the sensing device was further integrated with a portable and miniaturized potentiostat, leveraging Bluetooth technology for efficient data transmission. First, the paper-based microfluidic configuration was tested for optimal fluidic management to obtain optimized performance of the device. Subsequently, the two electrodes were individually tested to detect the two biomarkers, namely pH and Na^+^. The results demonstrated highly promising near-Nernstian (0.056 ± 0.002 V/dec) and super-Nernstian (− 0.080 ± 0.003 V/pH) responses, for Na^+^ and pH detection, respectively. Additionally, several important parameters such as storage stability, interferents, and memory effect by hysteresis study were also investigated. Finally, the butterfly-like paper-based microfluidic wearable device was tested for Na^+^ and pH monitoring during the physical activity of three volunteers engaged in different exercises, obtaining a good correlation between Na^+^ increase and dehydration phenomena. Furthermore, one volunteer was tested through a cardiopulmonary test, demonstrating a correlation between sodium Na^+^ increase and the energetic effort by the volunteer. Our wearable device highlights the high potential to enable early evaluation of dehydration and open up new opportunities in sports activity monitoring.

**Graphical abstract:**

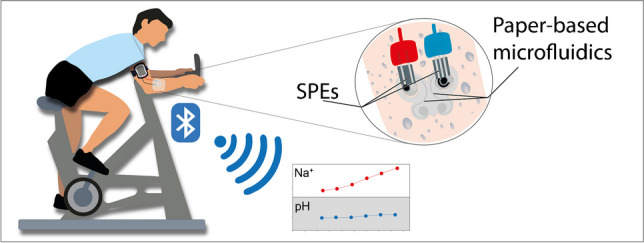

**Supplementary Information:**

The online version contains supplementary material available at 10.1007/s00604-024-06564-1.

## Introduction

Real-time patient health monitoring is a significant focus in current medical research. The goal is to enable self-monitoring of biomarkers directly by patients, allowing remote decentralized health monitoring at home as an alternative to traditional laboratory analyses. This transition from hospitals to a home-based healthcare approach is being facilitated by technological advancements, including wearable electrochemical (bio)sensors [1–3]. These sensors offer user-friendly and non-invasive analyses with easy data transfer and management [4, 5]. In particular, detecting chemical biomarkers in sweat provides a non-invasive and accessible method for monitoring health status during physical activity.

Significant progress has been made in this field, starting from the first pioneering works reported by Wei Gao et al. [6–8], which were mainly devoted to developing the sensing component and integrating the sensors with electronic platforms.

However, the reliable continuous sweat analysis requires addressing challenges in sampling owing to sweat secretion and reabsorption mechanisms [9]; thus, proper sweat transportation to the sensing element is needed to make the detection of the target analyte during the time, avoiding the mixing of sweat specimens [10–12]. For this reason, more recently, microfluidic-based sampling systems are gaining importance in delivering efficient wearable sensing tools for athletic activity monitoring [13–16]*.*

Nevertheless, PDMS/PMMA-based microfluidic channels are highly predisposed to trapping air bubbles [17–19]. These bubbles can cover the electrode’s surface and interfere with the real-time continuous monitoring of biomarkers in sweat.

To overcome this crucial issue, the paper represents a valuable alternative to the established PDMS-based microfluidic platforms thanks to its multifarious features. Indeed, the use of paper-based microfluidics is characterized by (i) sustainability and low cost, aligned with green analytical approaches [20]; (ii) capillary-driven pathways enabling the straightforward collection and flow transport without external pumps [21]; (iii) capability to act as a reservoir for reagent storing by delivering a reagent-free analytical tool; and (iv) eliminate the need for additional steps such as filtration or dilution, allowing for a sample treatment-free system [22–24]. Furthermore, the foldability of the paper and the electrochemical detection have recently opened a new route in microfluidics, paving the way for the sensing measurement based on origami configuration [25–29]. In recent studies, researchers have used paper for microfluidics in wearable sensor applications. Cao et al. exploited Whatman #4 filter paper to produce a vertical multilayer paper-based microfluidic patch integrated into a smartwatch sensor system [30]. This system detected Na^+^ and K^+^ by potentiometric measurements using modified screen-printed electrodes with the ranges of 4–20 mM for K^+^ and 42–60 mM for Na^+^ during stationary biking. Li et al. selected Whatman chromatography paper #1 to fabricate a foldable all-paper device for sweat analysis [31]. The paper-based device facilitated the collection, diffusion, and real-time analysis of lactate and glucose in sweat. Amperometric detection during physical activity yielded concentration curves for glucose and lactate in the ranges of 40–160 µM and 8–16 mM, respectively.

**Fig. 1:**
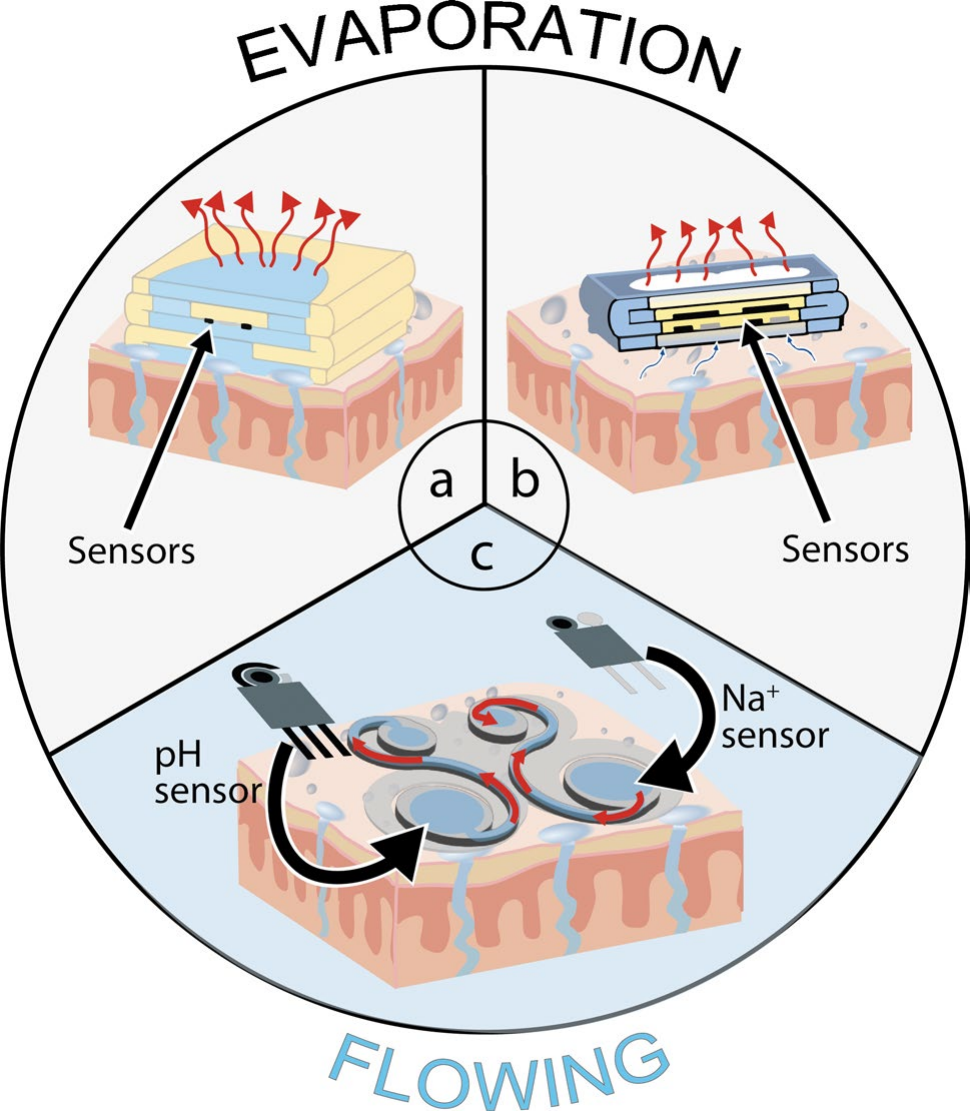
State of the art of paper-based microfluidic device to develop electrochemical wearable sensors for detection of (**a**) Na^+^ and K^+^ [30] and (**b**) glucose and lactate [31]. **c** Butterfly-like paper-based microfluidics developed in this work

If the paper-based vertical microfluidics, by adding the sample from the top to the bottom, allows to control (i) the volume to add and (ii) the flow rate, which is modulated by capillarity and gravity forces, in the case of origami configuration used for sweat sampling, the microfluidics goes in the opposite way, namely from the bottom to the up section, hindering control of the volume due to time and temperature-depending evaporation (Fig. 1a–b).

The process based on sample evaporation process on papers causes the preconcentration of the target analyte in the paper layers [32], and for the sweat analysis allows for the overestimation of the target analyte.

To manage this issue, we introduce an innovative butterfly-like paper-based microfluidic system for continuously monitoring pH and Na^+^ levels in sweat during physical activity based on potentiometric screen-printed electrodes. The butterfly-like configuration offers the absence of the evaporation and memory effects. The evaporation issue is managed by selecting lateral flow using a paper-based layer covered by a polyester film. The memory effect is overcome thanks to the instantaneous sampling of fresh sweat, which is then efficiently removed from the sensing zone, enabling precise and timely biomarker analysis in sweat (Fig. 1c).

This butterfly-like paper-based device enables further the simultaneous detection of the targeted biomarkers. This device was then integrated with a portable and miniaturized potentiostat, exploiting Bluetooth data transmission to deliver a wearable device tested on four volunteers during stationary biking activity, employing different workout programs. The results obtained were compared with reference methods, demonstrating the accuracy of this smart paper-based wearable multisensory developed.

## Experimental sections

### Reagents

4-tert-Butylcalix[4]arene (sodium ionophore X), 2-nitrophenyl octyl ether (O-NPOE), bis (2-ethylhexyl) sebacate (DOS), potassium tetrakis (4-chlorophenyl) borate (KTClPB), polyvinyl chloride (PVC), polyvinyl butyral (PVB), methanol, dimethylformamide (DMF), tetrahydrofuran (THF), sodium chloride, potassium chloride, magnesium chloride, calcium chloride, iridium chloride, hydrogen peroxide, oxalic acid, sodium carbonate, potassium ferricyanide, potassium dihydrogen phosphate, and dipotassium hydrogen phosphate were purchased from Sigma-Aldrich (USA). Carbon Black N220 (CB) was obtained from Cabot Corporation (Ravenna, Italy).

### Fabrication of screen-printed electrodes

Screen-printed electrodes were home-produced using a 245 DEK (Weymouth, UK) screen-printing machine. Flexible polyester films (Autostat HT5), purchased from Autotype Italia (Milan, Italy), were used as substrates to print the electrodes. Graphite ink (Electrodag 423 SS) from Acheson (Milan, Italy) was used to print the working electrode and the counter electrode (in the case of the pH sensor), while silver/silver chloride ink (Electrodag 6038 SS) was used to print the pseudo-reference electrode. Finally, a grey dielectric paste (D2070423P5) from Gwent Electronic Materials (Pontypool, UK) was used to print the insulating layer, to define the working electrode surface area. After each ink deposition, the electrodes were dried at 70 °C for 40 min for silver/silver chloride ink, 70 °C for 40 min for graphite-based ink, and 80 °C for 40 min for grey dielectric paste. The resultant diameter of the working electrode was 0.3 cm with a geometric area equal to 0.07 cm^2^.

### Preparation of the membranes for Na^+^ sensing

The sodium selective membrane was fabricated with the following compounds: 2.10% (w/w) KTClPB, 3.3% (w/w) sodium ionophore X, 30.9% (w/w), PVC, and 63.7% (w/w) DOS. Next, 1 mL for each 100 mg of solution of THF was added to the mixture and left to stir for 1 h with a magnetic stirrer. The reference membrane was prepared as follows: 79.1 mg of PVB and 50 mg of NaCl were dipped into 1 mL of methanol. The mixture was left under magnetic stirring for 1 h.

### Modification and conditioning of screen-printed electrodes for pH sensing

The working electrode was modified with iridium oxide by electrodeposition, carried out using cyclic voltammetry in iridium chloride solution by 60 scans in a range of potential comprised between 0 and 0.8 V, with a step potential of 0.05 V and a scan rate of 0.05 V/s. Next, electrodes were conditioned by applying a potential of 200 mV for 3 min in a pH 7 phosphate buffer [33].

### Modification and conditioning of screen-printed electrodes for Na^+^ sensing

Working and pseudo-reference electrodes were modified with the appropriate solutions using the drop-casting method deposition [34]. Firstly, 6 μL of CB dispersion was added onto the working electrode surface in three successive steps of 2 μL and left to dry for 1 h after each step. Then, 7.5 μL of sodium selective membrane was drop-cast onto the CB-modified working electrode and left to dry for 20 min. The pseudo-reference electrode was modified by casting 10 μL of the reference membrane solution and leaving it to dry for 20 min. Before the measurements, the ion-selective membrane was conditioned for 30 min with 10 µL of 1 M NaCl solution, while the reference membrane was conditioned for 18 h with 20 µL of KCl solution at 3 M. After, both membranes were carefully rinsed with 100 µL of distilled water.

### Fabrication of paper-based microfluidics

The microfluidic device was fabricated by a CO_2_ Laser Cutter VEVOR 40W, using Scottex® tissue paper as the substrate, while the pattern was drawn by Adobe Illustrator software. The microfluidic sample zone has a diameter of 10 mm, acting as a sweat collector. It is connected to the wasting zone by a channel of 3 mm in width. The wasting zone has a diameter of 8 mm.

### Electrochemical instrumentations

Potentiometric measurements for the optimization of pH and Na^+^ sensors were carried out by portable potentiostat Palmsens4 (Palmsens, The Netherlands). For simultaneous detection and on-body measurements, the “Emstat Pico development board” was used.

### pH and Na^+^ analysis in standard solution

Potentiometric measurements were carried out by dropping 80 μL of Britton-Robinson solution or NaCl solution for detection of pH and Na^+^ respectively, obtaining the signal in less than 30 s.

### Real-time on-body pH and Na^+^ measurement in sweat during physical activity using the wearable sensing device

On-body measurements were obtained during stationary biking of three volunteers. Emstat Pico development board was inserted in a commercially available case holder and inserted on the forearms of the subject. The system, consisting of the plastic protection system (namely polyester), the paper-based microfluidics, and the screen-printed electrodes, was securely attached to the epidermal surface using a Tegaderm™ patch.

### Measurements by reference methods

Analyses of sweat were carried out by an FC300B sodium ion electrode (Hanna instruments) and a pH60 VioLab benchtop pHmeter (XS instruments). Sweat was directly sampled by a microtube from the subject epidermis after the stationary biking activity.

### Cardiopulmonary exercise test

A cardiopulmonary test was carried out on a volunteer using the incremental exercise procedure, with the volunteer wearing the developed wearable analytical tool for Na^+^ and pH measurements. Simultaneously, a mask connected to specific instrumentation (Quark CPET, COSMED the metabolic company) was used for cardiopulmonary parameter detection.

## Results and discussion

The wearable device fabricated in this work encompasses the use of (i) a paper-based microfluidic device enabling punctual measurements in real-time and continuously during physical activity, (ii) two screen-printed electrodes for potentiometric measurement of pH and Na^+^ [33, 34], and (iii) a portable and miniaturized potentiostat module, Emstat Pico development kit, PalmSens [35] with a Bluetooth connectivity for data transmitting to a receiver.

A customized miniaturized sensing device has been developed to enable the recording of signals from both connected sensors, namely the electrochemical cell for pH detection and the electrochemical cell for Na^+^ detection, simultaneously.

**Fig. 2:**
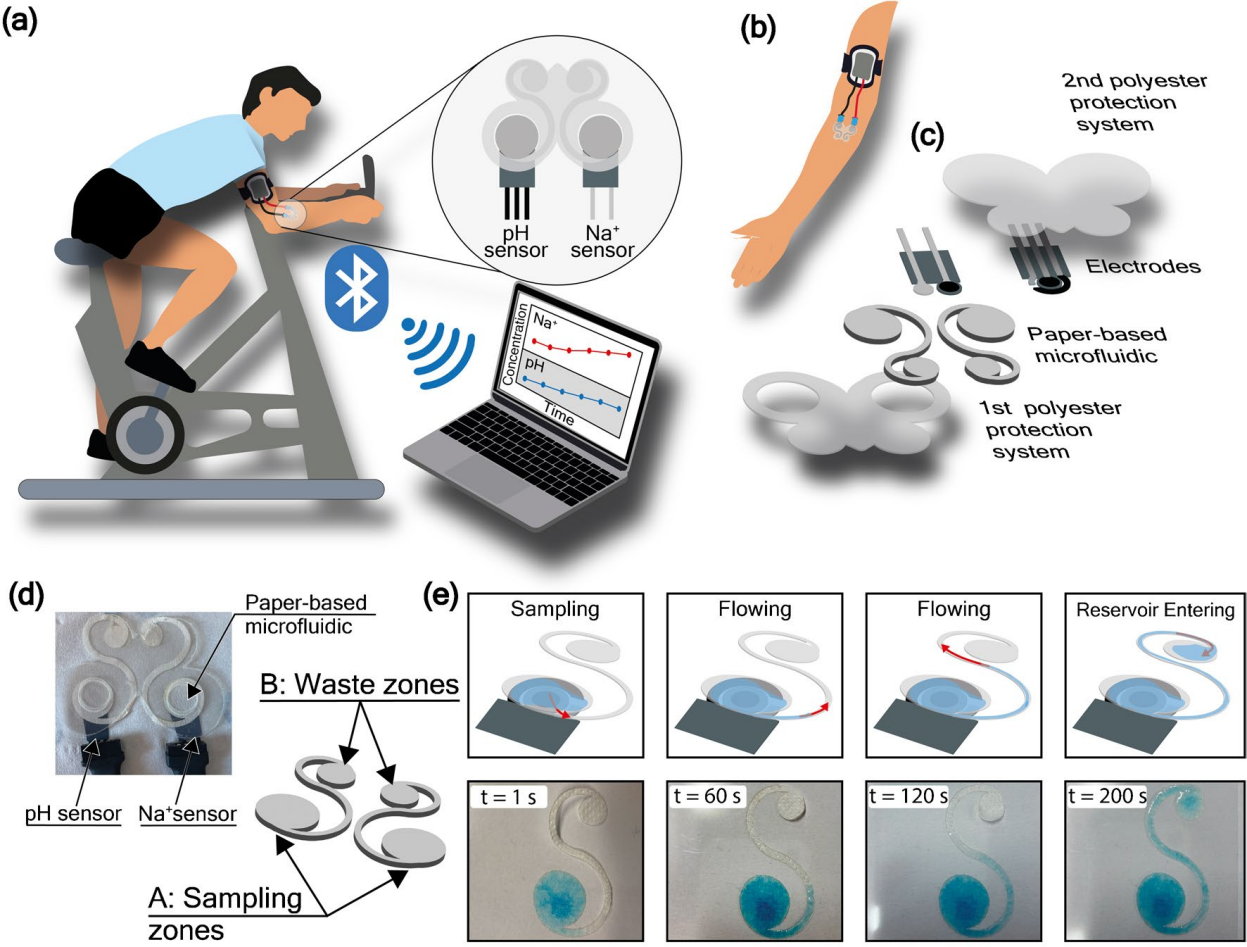
**a** Implementation of the developed wearable sensor on the forearm of a user during stationary biking, establishing a wireless connection to the laptop via Bluetooth for dehydration monitoring. **b** Set-up of the Emstat pico development board connected to the analytical device, inserted in a sportive holder to be worn on the forearm. **c** Extrude model of the Paper-based microfluidics and screen-printed electrodes setting during the real-time measurements. **d** Snapshot and design of the paper-based microfluidic device comprising the sampling and waste zones. **e** Investigation of paper-based microfluidic sampling and flowing behavior using a solution of 1 mM methylene blue on one wing of the butterfly microfluidics

To demonstrate that the paper-based microfluidic overcomes the memory effect and avoids preconcentration due to uncontrolled evaporation, the two potentiometric sensors were independently optimized and tested separately with a paper-based microfluidics system. Finally, the sensing device has been integrated with the Emstat Pico development board (Fig. 2). The potentiostat module and the wire connections have been customized to deliver the prototype for the simultaneous detection of pH and Na^+^ during physical activity to compare with data obtained with the reference methods.

### Butterfly-like paper-based microfluidics

The paper microfluidic device was fabricated by engraving a tissue paper substrate (i.e., Scottex® paper) by a CO_2_ laser machine to develop a butterfly-like shape. Each of the two wings will be placed in contact with the pH and Na^+^ sensor, respectively, for the potentiometric measurement (Fig. 2a–c). In detail, the function of the paper microfluidic device relies on the presence of two different areas, namely the sampling zones and the waste zones (Fig. 2d). The sampling zones act as a collector for the produced sweat and the sensing zone for the analysis, being integrated with the electrode for the potentiometric measurement. Once the sweat is analyzed, it is automatically delivered to the waste zones, where an adsorbent pad adsorbs the sample through a connection channel (2 mm in width), acting both as a passive pump and a waste reservoir. The shape of both zones and the connection channel were examined by flowing the collected sample throughout one wing of the butterfly microfluidic device. This configuration enables the collection of newly produced sweat, avoiding the memory effect, and delivering the accurate continuous monitoring of the target analytes because microfluidics is not based on evaporation but only on capillary force.

Firstly, we tested the time to manage the fluid. Thus, we introduced a methylene blue probe solution into the device. At regular intervals of 60 s, 30 µL of a solution containing methylene blue at 1 mM were deposited onto the sampling zone. As shown in Fig. 2e, the sampling zone promptly absorbed the sample through capillary action, evidenced by the blue coloration. The capillary forces then facilitated the movement of the solution through the paper channels to the waste zone, creating a continuous flow that allowed newly produced sweat to enter the sampling zone for subsequent analysis.

After, the butterfly-like paper-based microfluidics was tested with electrochemical devices, previously independently optimized.

### pH sensing

The electrochemical sensor for pH measurements was fabricated following the protocol developed in our previous work [33], where we exploited the unique properties of iridium oxide as an H^+ ^sensitive layer to produce a highly effective Kapton-based potentiometric sensor for pH monitoring in sweat during physical activity. Considering our previous study, we used the optimized protocol to electrodeposit the iridium oxide layer on the polyester-based graphite working electrode. The produced iridium oxide is sensitive to H^+^ ions in solution, following a mechanism that relies on the dependence of the iridium oxidation state upon the number of hydrogen ions present in an aqueous solution, according to the following reactions [36]:

$${2\text{IrO}}_{2}+{2\text{H}}^{+}+{2\text{e}}^{-}\leftrightarrow {\text{Ir}}_{2}{\text{O}}_{3}+{\text{H}}_{2}\text{O}$$

$$2{\left[{\text{IrO}}_{2}{\left(\text{OH}\right)}_{2}\cdot {2\text{H}}_{2}\text{O}\right]}^{2-}+{3\text{H}}^{+}+{2\text{e}}^{-}\leftrightarrow {\left[{\text{Ir}}_{2}{\text{O}}_{3}{\left(\text{OH}\right)}_{3}\cdot {3\text{H}}_{2}\text{O}\right]}^{3-}+{3\text{H}}_{2}\text{O}$$

**Fig. 3:**
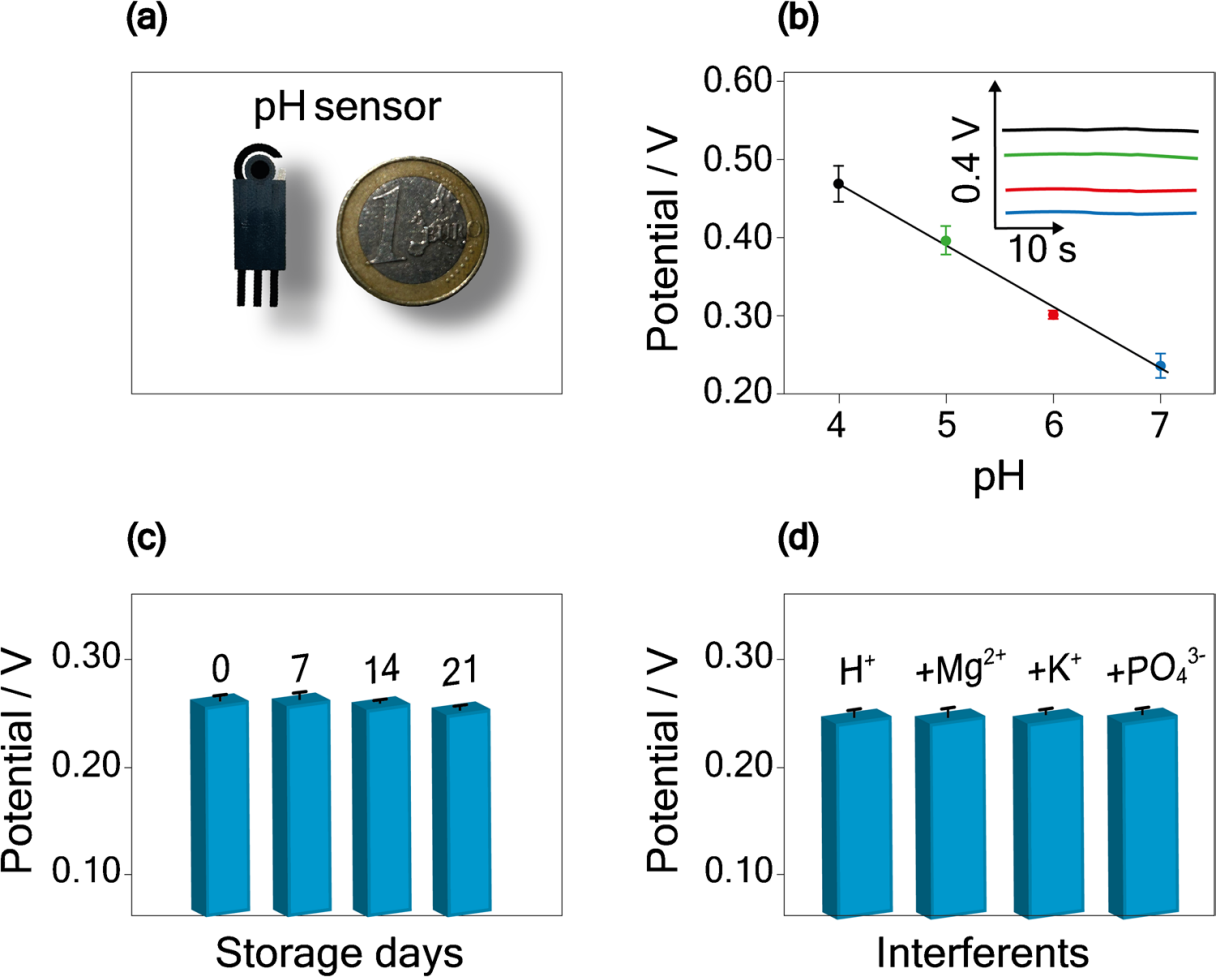
**a** Picture of the screen-printed electrode for the pH potentiometric sensing. **b** Calibration curve obtained by measuring 80 µL of Britton-Robinson buffer solution with a pH range of 4 to 7. Insets: potentiometric response. **c** Histogram bars obtained for the detection of Britton-Robinson buffer with a pH of 7 on the same day of the iridium oxide electrodeposition, and after 7, 14, and 21 days (electrodes are stored in a vacuum, at − 18 °C). **d** Histogram bars obtained for the detection of Britton-Robinson buffer with a pH of 7, in the presence of 18 mM K^+^, 0.082 mM Mg^2+^, and 0.31 mM PO_4_^3−^

Since the pH physiological value in sweat falls between 5 and 7 [37], we first focused on the analyses of pH standard solution within this specific range. A linear correlation between pH value and potential was obtained between 4 and 7, with a calibration curve equation equal to *y* = (− 0.080 ± 0.003) *x* + (0.79 ± 0.02), *R*^2^ = 0.980, with *y* the potential and *x* the pH values (Fig. 3b). When analyzing a Britton-Robinson buffer at a pH of 7, the inter-electrode reproducibility was found to be 3% RSD (Relative Standard Deviation), with a sample size of *n* = 3.

The recorded super-Nernstian response, characterized by a slope of (− 0.080 ± 0.003) V/pH, is a direct result of the iridium oxide layer composition in the system. This response is usually obtained when using this particular system [38, 39]. Compared to the sensitivity obtained when using the Kapton substrate, namely (− 0.079 ± 0.002) V/pH, we observed a comparable value within the experimental error using a cost-effective and sustainable substrate, namely polyester. This finding demonstrates the robustness of the fine-tuned procedure utilized in this study.

### Storage stability

Considering the application of the pH sensor in a wearable device, a crucial aspect to examine is the storage stability of the developed sensor over time. To address this concern, pH potentiometric measurements were carried out on the same day of the iridium oxide electrodeposition, and then at intervals of 7, 14, and 21 days. The electrodes were stored at − 18 °C under vacuum conditions during these periods to assess their performance and durability over an extended storage duration. When measuring Britton-Robinson buffer at pH 7, the results shown in Fig. 3c highlight a stable signal within the investigated temporal range, with a potential decrease of 1.2% after 21 days from the initial iridium oxide electrodeposition.

The results shown in Fig. 3c report high stability of the printed electrochemical potentiometric sensors and low RSD % during the days (from 3% day 0 to 1% day 21). This behavior can probably be ascribed to the uniformity of the conditions used to store the screen-printed electrodes before carrying out the measurements. The uniform, controlled, and constant conditions of the iridium oxide sensing layer are probably responsible for the slight RSD % decrease, as we observed in our previous work using Kapton as support to print the electrochemical sensor [33].

### Interference study

To evaluate the selectivity of the pH sensor in the presence of interfering ions usually found in sweat samples, potentiometric measurements were carried out by testing Britton-Robinson buffer at pH 7, in the presence of 18 mM K^+^, 0.082 mM Mg^2+^, and 0.31 mM PO_4_^3−^. As depicted in Fig. 3d, the recorded potential exhibited a negligible contribution in the presence of these ions, validating the good selectivity of the sensor. Indeed, iridium oxide-based pH sensors are free from interference from various simple ions and complexing agents, further underscoring their reliable performance in practical applications [39, 40].

### Na^+^ sensing

For the development of the Na^+^ sensor, we used a working electrode surface modified with carbon black nanomaterial to obtain a stable and reproducible signal and an ionophore-containing membrane to recognize the Na^+^ target analyte [34] selectively. The reference electrode was modified with a PVB-based solution (reported in the literature [6]), to obtain enhanced stability, a condition strictly required for long-time analyses.

Furthermore, considering the use of the Na^+^ sensor for the application in the wearable device for real matrix analysis, we investigated the composition of the ion-selective membrane and the use of a conditioning process on this membrane. The conditioning process is a procedure performed to minimize the potential drift, occurring when performing long-time analysis [6]. This process is crucial when referring to the use of wearable sensors where a long-time measurement is needed (i.e., greater than 40 min). Two different conditioning parameters for the ion-selective membrane were evaluated, namely the time of the process and the concentration of the NaCl conditioning solution (Table S1). Specifically, 10 μL of NaCl solution at different concentrations was used to condition the ion-selective membrane for different periods before the measurements. The sensitivity of the calibration curves obtained by varying the conditioning time from 10 min to 3 h, and the NaCl concentration from 10^−6^ M to 3 M, did not show a Nernstian response (Table S1). For this reason, we decided to change the O-NPOE plasticizer used previously with the DOS plasticizer. Indeed, the use of a different plasticizer can actively influence the performances of the sensors, such as the Nernstian slope, and selectivity [41].

**Fig. 4:**
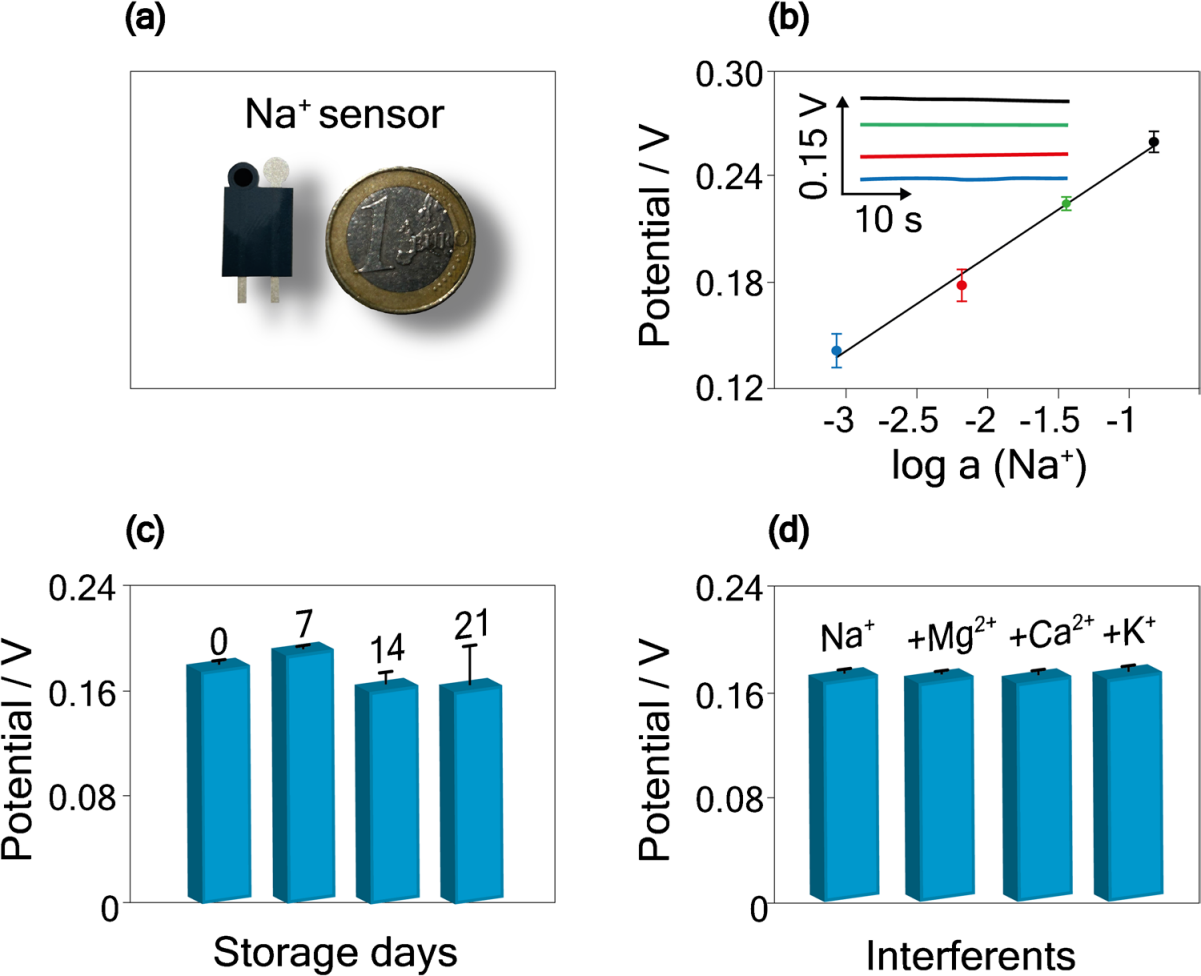
**a** Picture of the screen-printed electrode for the Na^+^ potentiometric sensing. **b** Calibration curve obtained for the detection of Na^+^ in the concentration range of 0.001–1 M. Insets: potentiometric response. **c** Histogram bars obtained for the detection of 0.01 M Na^+^ on the same day of the ion-selective and reference membranes deposition, and after 7, 14, and 21 days. **d** Histogram bars obtained for the detection of 0.01 M Na^+^ in the presence of 18 mM K^+^, 1 mM Ca^2+^, and 0.5 mM Mg^2+^

Using the DOS-based ion-selective membrane, we first evaluated the solution concentration used to condition the ion-selective membrane cast onto the working electrode. Specifically, 10 μL of NaCl solution at different concentrations, namely 10^−3^ M, 10^−1^ M, 1 M, and 3 M, was used to condition the ion-selective membrane for 30 min before the measurements. A value closer to a Nernstian behavior, namely (0.054 ± 0.003) V/dec, was observed with a NaCl conditioning solution concentration of 1 M; thus, this concentration was adopted for the rest of the work. Furthermore, the time of the conditioning step was also evaluated by investigating the response obtained after conditioning the ion-selective membrane for 5 min, 10 min, 30 min, 1 h, and 3 h, with a solution of 1 M NaCl. After the conditioning step of 30 min, a slope closer to a Nernstian behavior equal to 0.055 ± 0.002 V/dec was obtained. The conditioning step of a 1 M NaCl solution for 30 min was then selected and a calibration curve was obtained with Na^+^ concentration up to 1 M. A linear correlation between potential and Na^+^ concentration was obtained, described by the calibration curve equation equal to *y* = (0.056 ± 0.002) *x* + (0.300 ± 0.004), *R*^2^ = 0.993, with *y* is the potential and *x* is the log a (Na^+^) (Fig. 4b). The inter-electrode reproducibility when analyzing a solution containing 0.01 M Na^+^ was equal to 2% RSD (*n* = 3).

### Storage stability

Storage stability was investigated by measuring 0.01 M Na^+^ on the same day of the ion-selective and reference membrane deposition, and after 7, 14, and 21 days, storing the electrodes at room temperature, under vacuum conditions. As depicted in Fig. 4c, a slight decrease in the potential was recorded after 14 days, equal to a decrease of 11%. However, the signal remained stable up to 21 days from the membrane deposition, comparable with the one reported in the literature [36, 40].

### Interference studies

With the aim to evaluate the selectivity of the Na^+^ sensor toward the presence of interfering ions usually found in sweat samples [42], measurements were carried out by testing a solution of Na^+^ at 0.01 M, in the presence of 18 mM K^+^, 1 mM Ca^2+^, and 0.5 mM Mg^2+^. As depicted in Fig. 4d, a negligible contribution by the presence of these compounds was observed, in agreement with the literature, where a non-significative signal was recorded [6, 30].

### Evaluation of the butterfly-like paper-based microfluidics for electrochemical detection of Na^+^ and pH

**Fig. 5:**
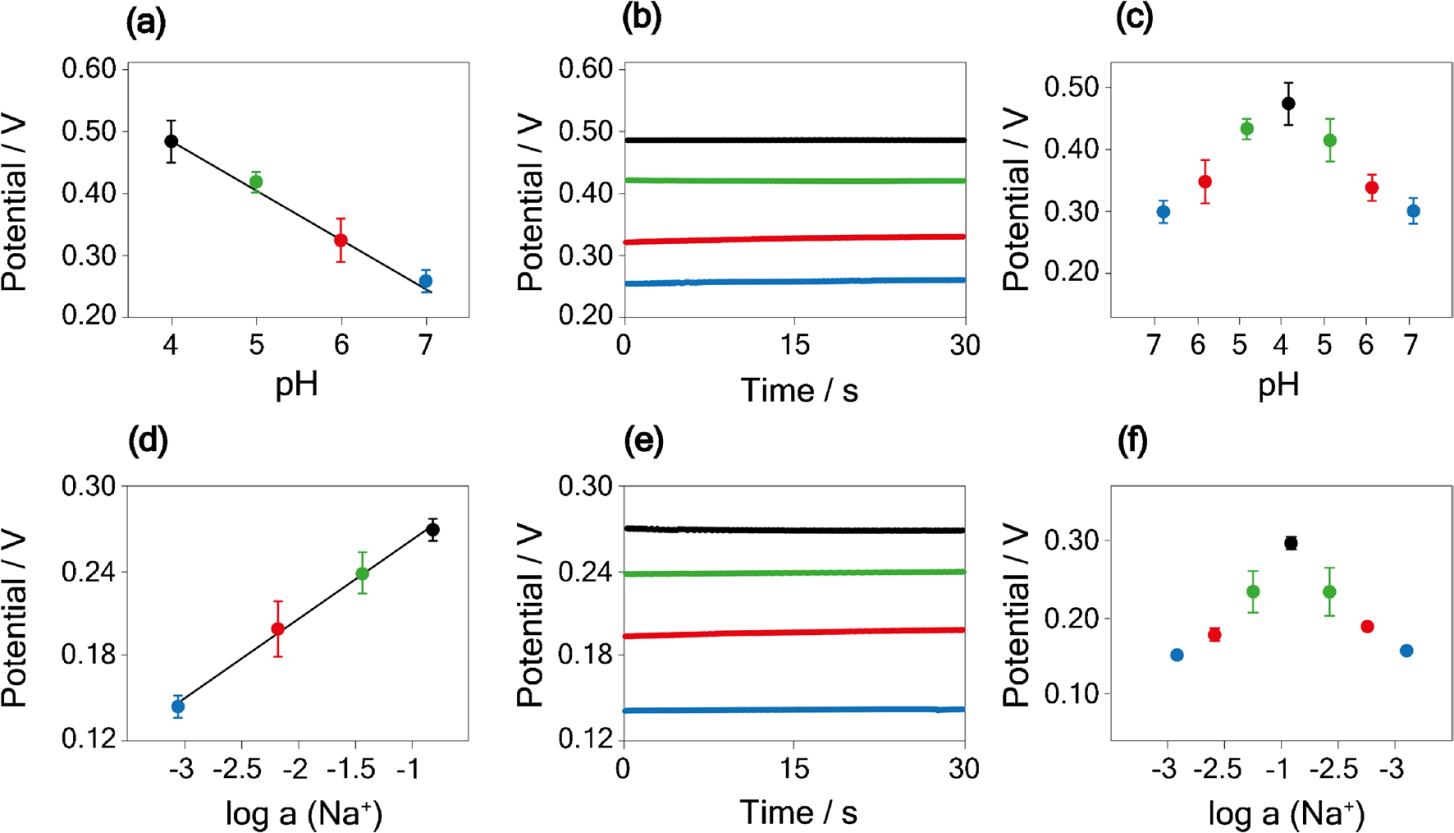
**a** Calibration curve and **b** relative potentiograms obtained using the screen-printed electrodes integrated paper-based microfluidic device, measuring 80 µL of Britton-Robinson buffer solution in the pH range of 4–7. **c** Hysteresis study for the pH sensor measuring 80 µL of Britton-Robinson buffer solution in the pH range of 4–7 and vice versa. **d** Calibration curve and **e** relative potentiograms obtained using the screen-printed electrodes integrated paper-based microfluidic device, measuring 80 µL of Na^+^ standard solution in the range of 0.001–1 M. **f** Hysteresis study for the Na^+^ sensor measuring 80 µL of Na^+^ standard solution in the range of 0.001–1 M and vice versa

To investigate the efficiency of the developed paper-based microfluidics, the device was then used for the potentiometric measurements of both analytes. First, the microfluidic platform was carefully placed on the surface of the sensors, ensuring the sampling zone was in contact with the modified electrodes. Subsequently, a calibration curve was obtained by adding 20 µL of the selected analyte at different concentrations, directly on the sampling zones placed onto the corresponding sensor. In detail, using the sensor developed for pH detection, the calibration curve was first obtained by analyzing the Britton-Robinson buffer solution in the pH range between 4 and 7. A linear correlation was described by the equation *y* = (− 0.080 ± 0.004) *x* + (0.81 ± 0.02), *R*^2^ = 0.983, with *y* the potential and *x* the pH value (Fig. 5a–b). Noteworthy, compared to the calibration curve obtained in the absence of the paper-based microfluid, the use of the paper microfluidic device showed no significant variation in the recorded slope. The inter-electrode reproducibility calculated by analyzing a pH 5 solution was equal to 4% RSD (*n* = 3). Subsequently, the paper-based microfluidics system was tested for the detection of Na^+^ in a standard solution at a concentration equal to 10^−3^ M, 10^−2^ M, 10^−1^ M, and 1 M. A linear correlation between the potential and the activity was obtained, represented by *y* = (0.056 ± 0.005) *x* + (0.320 ± 0.009), *R*^2^ = 0.961 (Fig. 5d–e). Similar to the calibration curve obtained without the paper-based microfluidics, the use of this configuration demonstrated no significant variation in the recorded slope. The inter-electrode reproducibility calculated by analyzing a solution containing 0.01 M Na^+^ exhibited 3% RSD with a sample size of *n* = 3.

To prove that this device faces the memory effect, we conducted a hysteresis study on both pH and Na^+^ sensors. Figure 5c illustrates the pH detection in the range of 4 to 7 and vice versa using the same electrodes. Figure 5f depicts the hysteresis study of the Na^+^ sensor, with Na^+^ concentration varying from 0.001 to 1 M and back. Both developed sensors showed only a slight hysteresis effect, allowing accurate analyte measurements without any memory effect.

### Assessment of simultaneous detection of Na^+^ and pH in standard solution using the butterfly-like paper-based microfluidic electrochemical device

For the accuracy evaluation during on-body analyses, the device was miniaturized by combining the paper-based microfluidic device and the screen-printed electrodes with the EmStat Pico development board. The response of the integrated device was evaluated by calibration curves in the standard solution (Fig. S1). For pH detection, a sensitivity equal to (− 0.084 ± 0.004) V/pH was obtained, consistent with the value obtained in the presence of the microfluidic device using portable potentiostat Palmsens4 (i.e., − 0.080 ± 0.004 V/pH). For Na^+^ detection, a sensitivity equal to 0.061 ± 0.002 V/dec was obtained, consistent with the value obtained previously (i.e., 0.056 ± 0.005 V/dec), considering the experimental error, demonstrating the reliability of the miniaturized transducer.

### Wearable system for continuous on-body monitoring of pH and Na^+^ during stationary biking

To enable continuous on-body monitoring of pH and Na^+^, the flexible sensors were in contact with volunteers’ epidermis through our paper-based microfluidic device to facilitate the collection of produced sweat and the removal of old sweat. In detail, the first layer of polyester (Fig. 2c) was used to put in contact only the sampling zone with the epidermis, while the second layer of polyester (Fig. 2c) was used to protect the sensors and the paper microfluidic device as well as to avoid the sweat evaporation. A final Tegaderm patch was used to hold the whole device to the body firmly. The miniaturized Emstat Pico development board was inserted in a commercially available holder for running activity and placed on the forearm of the volunteer.

The potentiometric values were transmitted to a laptop for data analysis during 40-min stationary biking of 3 volunteers. Specifically, concerning the pH measurements during the physical activity, no significant variation in the values was observed throughout the cycling duration. However, for sodium detection, a different behavior was recorded among subjects, depending on exercise intensity levels.

**Fig. 6:**
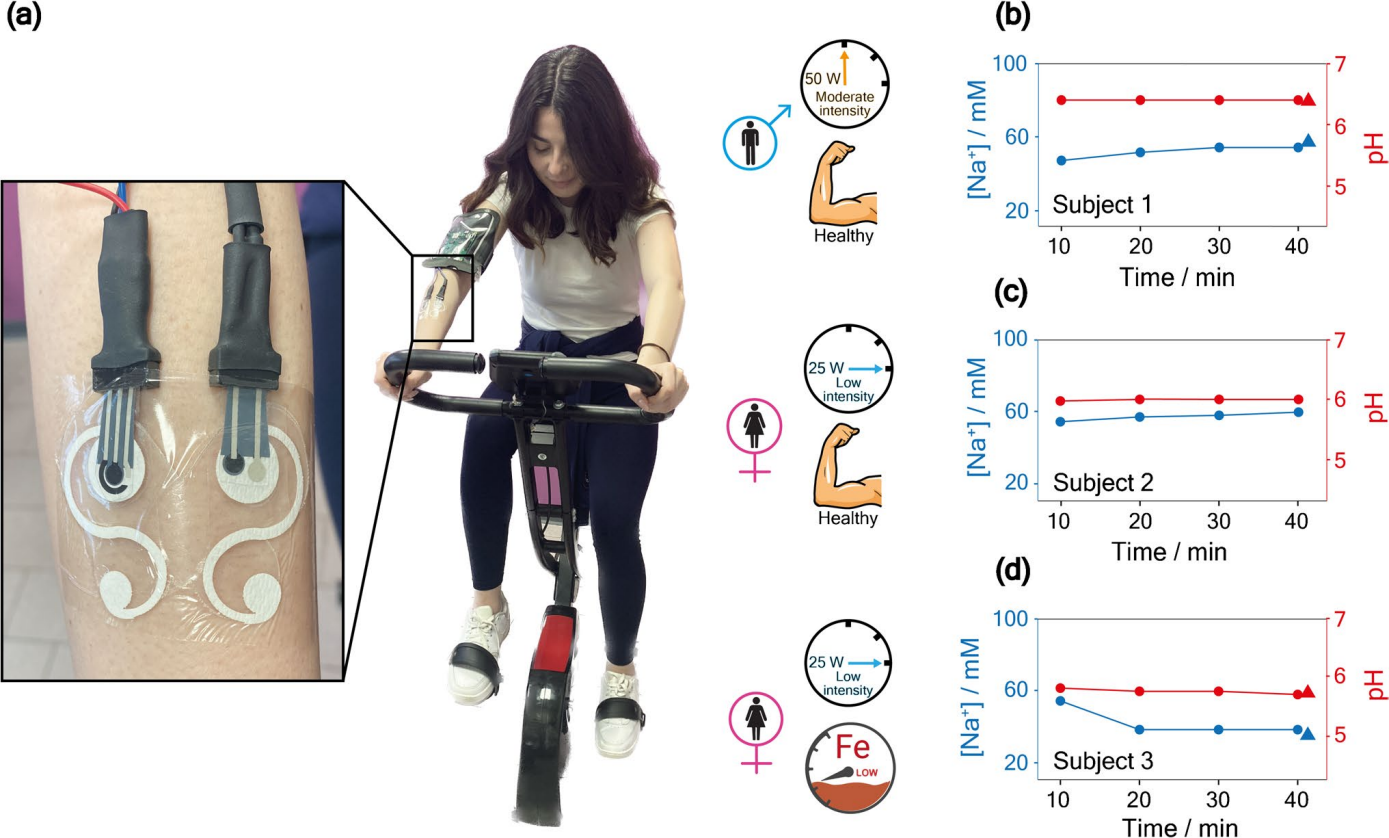
**a** Snapshot of the wearable set-up for Na^+^ and pH measurements during stationary biking exercise, and relative concentration profile obtained on **b** subject 1, **c** subject 2, and **d** subject 3. Symbols: circles represent measurements carried out by our wearable sensing tool; triangles represent measurements carried out by reference methods

For male subject number one, a medium workout program in the stationary biking software was used, with a constant power of 50 Watt (Fig. 6a). This workload required serious effort for each pedal stroke due to its moderate intensity. A slight increase in the sodium concentration from 47 to 55 mM after 40 min of cycling was recorded, highlighting the presence of moderate dehydration phenomena (Fig. 6b). This observation can be ascribed to the subject’s strong energetic effort during the physical activity.

For female subject number two, a low-intensity workout program was used, with a constant power of 25 Watt. During the time of the exercise, we did not record any significant variation in sodium concentration for this subject (Fig. 6c). This can probably be ascribed to the absence of dehydration phenomena and low sweat rate due to the light energetic effort of the volunteer.

Female subject number three (affected by iron deficiency) was tested under low-intensity exercise too. A decrease in the sodium concentration was recorded after the first 10 min (from 52 to 35 mM), followed by a relatively stable value maintained between 20- and 40-min intervals of physical activity (Fig. 6d). This is likely due to the combination of iron deficiency affecting the subject and low energetic effort during the physical activity, which may result in a slower sweat rate and reduced occurrence of dehydration phenomena. The analyses performed using the reference methods based on bulk ion-selective electrodes (Fig. 6b and d, triangle symbols) demonstrated significant agreement with the values obtained from our newly developed wearable analytical tool (circular symbols), highlighting the accuracy of this device. The comparison was possible for sweat collected from subjects 1 and 3 at the end of the performance for the higher volume required using the reference methods. The comparison at the end of the physical excise, i.e., 40 min, allows to have more time to properly evaluate the evaporation effect. In the case of subject 2, the amount was insufficient for analysis using the reference methods, demonstrating the advantages of using the wearable device not only for continuous monitoring but also for low volume sample required.

Compared to the wearable sensors reported in the literature for sweat analysis (Table S2), the herein-developed paper-based microfluidic device has similar analytical features in detecting the indicated target analytes, considering the obtained linear ranges and sensitivity. However, the PDMS-based microfluidics reported in Table S2 can have the issue of air bubbles [17–19], which cover the electrode’s surface and interfere with real-time continuous monitoring of biomarkers in sweat. Furthermore, PDMS-based microfluidic production requires a more complex setup than paper-based microfluidic systems. Thus, the herein-developed paper-based microfluidic based on a lateral flow ensures the absence of the issue of air bubbles and requires a low-cost and easy-to-use setup for the fabrication, supplying accurate measurements without any memory effect or evaporation issues.

### Comprehensive monitoring of pH, Na^+^, and energetic effort during cardiopulmonary test

**Fig. 7:**
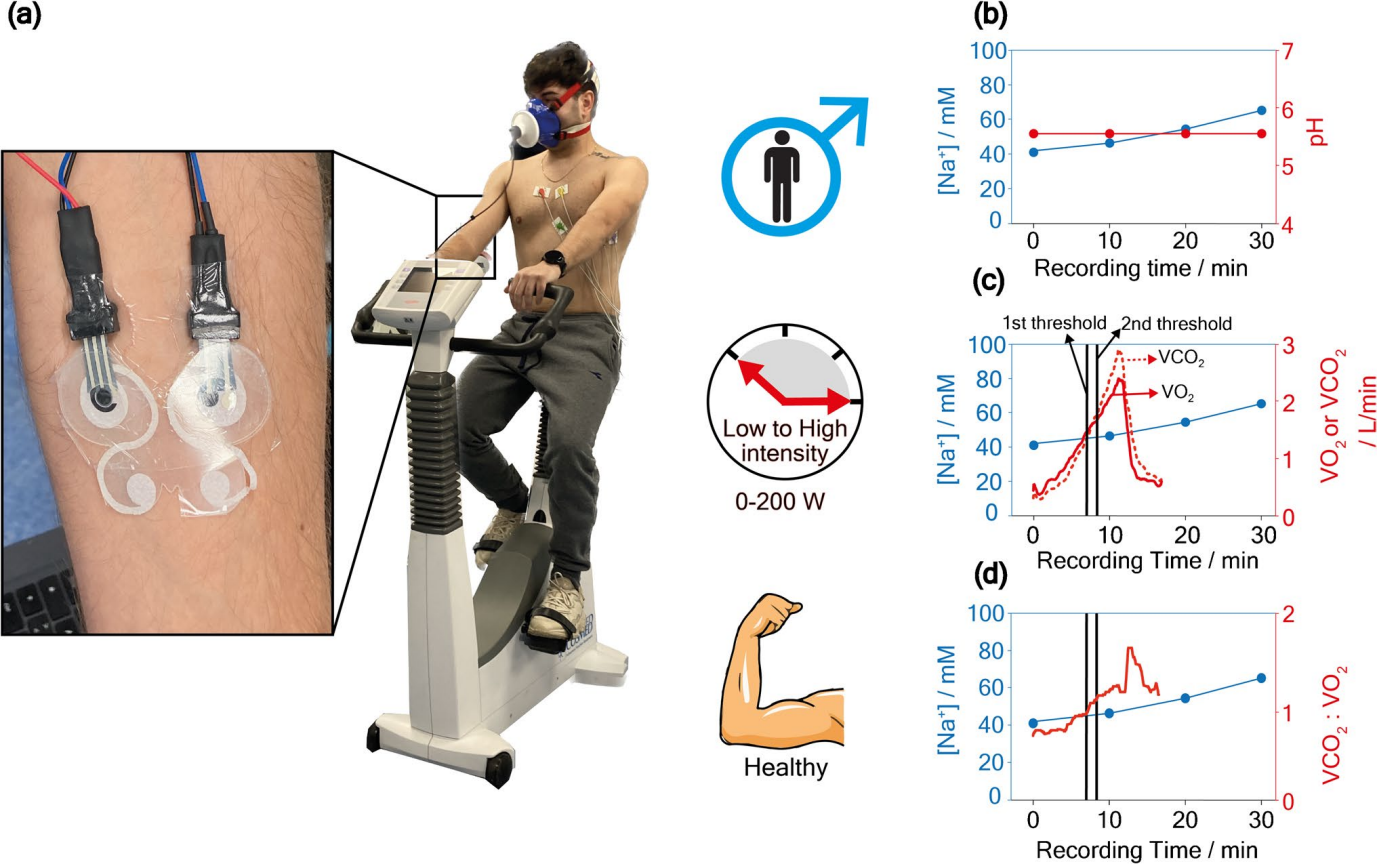
**a** Snapshot of the wearable set-up for Na^+^ and pH measurements during the cardiopulmonary test. **b** Relative concentration profile obtained on subject 4. **c** Overlap of Na^+^ profile with oxygen uptake (red solid line) and the produced CO_2_ (red dashed line) obtained during the cardiopulmonary test. **d** Overlap of Na^+^ profile with the ratio of VCO_2_:VO_2_ obtained during the cardiopulmonary test

To evaluate the applicability in sports medicine, we examined the connection between volunteers’ energetic effort and the sodium variation during their physical activity, a male subject underwent a standardized cardiopulmonary exercise test on medical stationary biking equipment (Fig. 7a) [43]. This instrumentation provides an accurate workout program, requiring the athlete to exert a strong effort to cycle, with power intensity incrementing from 0 to 200 Watt. Noteworthy, this medical tool can capture various data during incremental physical exercise, such as the breath exhaled per minute (Ve), the oxygen uptake (VO_2_), and the CO_2_ (VCO_2_) production. The acquisition of these data enables the calculation of an important parameter known as the anaerobic threshold. This parameter is closely connected to the production of CO_2_ and consumption of O_2_ during exercise and is observed when the contribution of the anaerobic phase is dominant in the overall metabolism [44].

For the execution of the cardiopulmonary test, the subject first underwent a warm-up exercise, i.e., a low-intensity cycling activity for 10 min. Following the warm-up, the cardiopulmonary exercise test was initiated. As shown in Fig. 7c, the first anaerobic threshold was recorded after 7.0 min of exercise, highlighted by the increasing slope of the VO_2_ profile shown in the solid red line, i.e., increased O_2_ consumption. At 8.2 min, a second anaerobic threshold was observed as depicted by the dashed red line, indicating an increase in the VCO_2_ slope accompanied by elevated CO_2_ production and exhalation. Both parameters reached their peak values after 13 min, with VO_2_ and VCO_2_ values reaching 2420 ml/min and 2945 ml/min, respectively. The respiratory exchange ratio (RER), represented by the VCO_2_/VO_2_ ratio, was also assessed as an important parameter to evaluate the exercise effort (Fig. 7d).

When the exercise intensity increases, the RER is higher as a result of the faster rise in VCO_2_ compared to VO_2_ [45]. In order to obtain an optimal high-exercise effort, a ratio higher than 1.15 must be reached. For the subject under evaluation, the ratio reached 1.62 after 13 min of exercise, highlighting the increase of CO_2_ production that overtakes the consumption of O_2_. Comparing the Na^+^ profile with the VO_2_ and VCO_2_ data, an increase in the Na^+^ concentration after the second anaerobic threshold was obtained, i.e., from 41 up to 67 mM after 30 min. This can be attributed to the subject’s vigorous exertion during the physical activity, producing a high sweat rate and dehydration process, which takes place only after the second threshold, when a high CO_2_ amount is exhaled by the breath (Fig. 7c). However, we observed the increase in the Na^+^ concentration was slightly delayed compared to the occurrence of the first and second thresholds. Furthermore, even after the completion of the cardiopulmonary exercise test, the Na^+^ profile continued to be recorded for up to 30 min, providing valuable insights into the subject’s post-exercise recovery period.

Finally, the results obtained by subject 4 (i.e., incremental heavy workout) are compared with those obtained by subjects 1, 2, and 3 (i.e., low or moderate workout). The results indicated that the variation in the Na^+^ profile can offer a preliminary evaluation of the physiological response related to workout programs, enabling the differentiation between a light and an intense program, when the anaerobic threshold is usually reached [46].

## Conclusions

In pursuit of a reliable wearable analytical device, we successfully designed an electrochemical sensing platform capable of continuous monitoring of Na^+^ and pH during stationary biking activity. Our system leverages custom-made screen-printed electrodes for potentiometric measurements of both analytes, along with a butterfly-like paper-based microfluidic device for sweat sampling and biomarker analysis in sweat. Additionally, we incorporated a portable and miniaturized potentiostat for data acquisition and transmission through Bluetooth technology.

Furthermore, by employing different workout program intensities and performing a cardiopulmonary exercise test, the device revealed that low-energy workouts resulted in slight Na^+^ and pH concentration variations, indicating an absence of dehydration phenomena. Conversely, high-energy workouts triggered dehydration, leading to increased Na^+^ concentration in sweat, while pH remained relatively stable. Leveraging the electrochemical features of our screen-printed electrodes, the properties of the butterfly-like paper-based microfluidics, and the miniaturized potentiostat, our wearable electrochemical sensor will be tested during a high number of sports exercises to further demonstrate to be a viable non-invasive and user-friendly analytical tool for dehydration evaluation, by improving the time of analysis through the increase of the length in the configuration and the dimension of adsorbent pad. Our work can be exploited to transition hospital-based healthcare systems and laboratory-based analyses to a more accessible home-based healthcare approach—a promising step forward in improving overall health monitoring and personalized care.

## Supplementary Information

Below is the link to the electronic supplementary material.

Supplementary file1 (DOCX 132 KB)

## Data Availability

The datasets generated during and/or analyzed during the current study are available from the corresponding author on reasonable request.
