# Supplementary material for: Wearable electrochemical device based on butterfly-like paper-based microfluidics for pH and Na+ monitoring in sweat
Source: Mikrochim Acta. 2024 Sep 7;191(10):580. doi: 10.1007/s00604-024-06564-1 (PMC11380643; doi:10.1007/s00604-024-06564-1)
Supplement: Supplementary file 1 — Supplementary file1 (DOCX 132 KB) [file 604_2024_6564_MOESM1_ESM.docx]

**Wearable electrochemical device based on butterfly-like paper-based microfluidics for pH and Na^+^ monitoring in sweat**

Luca Fiore ‡^a,b^, Vincenzo Mazzaracchio‡^a^, Arianna Antinucci ^a^, Roberto Ferrara ^c^, Tommaso Sciarra ^c,d^, Florigio Lista ^d^, Amy Q. Shen ^e^, and Fabiana Arduini*^a,b^

^a^ Department of Chemical Science and Technologies, University of Rome Tor Vergata, Via della Ricerca Scientifica 1, 00133, Rome, Italy.

^b^ SENSE4MED, via Bitonto 139, 00133, Rome, Italy.

^c^ Physical Medicine and Rehabilitation Unit, Italian Army Medical Hospital, 00184 Rome, Italy.

^d^ Defence Institute for Biomedical Sciences. Rome, Italy

^e^ Micro/Bio/Nanofluidics Unit Okinawa Institute of Science and Technology Graduate University 1919-1 Tancha, Onna-son, 904-0495, Okinawa, Japan

† These authors contributed equally.

*Corresponding author. Department of Chemical Science and Technologies, University of Rome Tor Vergata, Via della Ricerca Scientifica 1, 00133, Rome, Italy. E-mail: Fabiana.arduini@uniroma2.it

**CONTENTS**

**Table S1** Evaluated parameters for the conditioning of the O-NPOE-based ion-selective membrane

| **Conditioning time** | **[NaCl] (M)** | **Slope (V/dec)** |
| --- | --- | --- |
|  |  |  |
| 10 min | 1 | 0.065 |
|  |  |  |
| 30 min | 10^-6^ | 0.080 |
|  | 10^-5^ | 0.067 |
|  | 10^-4^ | 0.072 |
|  | 10^-3^ | 0.077 |
|  | 10^-1^ | 0.085 |
|  | 1 M | 0.075 |
|  | 3 M | 0.077 |
|  |  |  |
| 1 h | 10^-5^ | 0.080 |
|  | 1 M | 0.066 |
|  |  |  |
| 3 h | 1 M | 0.071 |
|  |  |  |

**Figure S1.** Potentiogramms obtained for pH (a) and Na^+^ (b) detection and relative calibration curves (c and d) performed in standard solutions, by using the integrated sensing device with the paper-based microfluidic device, the SPEs, and the EmStat Pico development board.

**
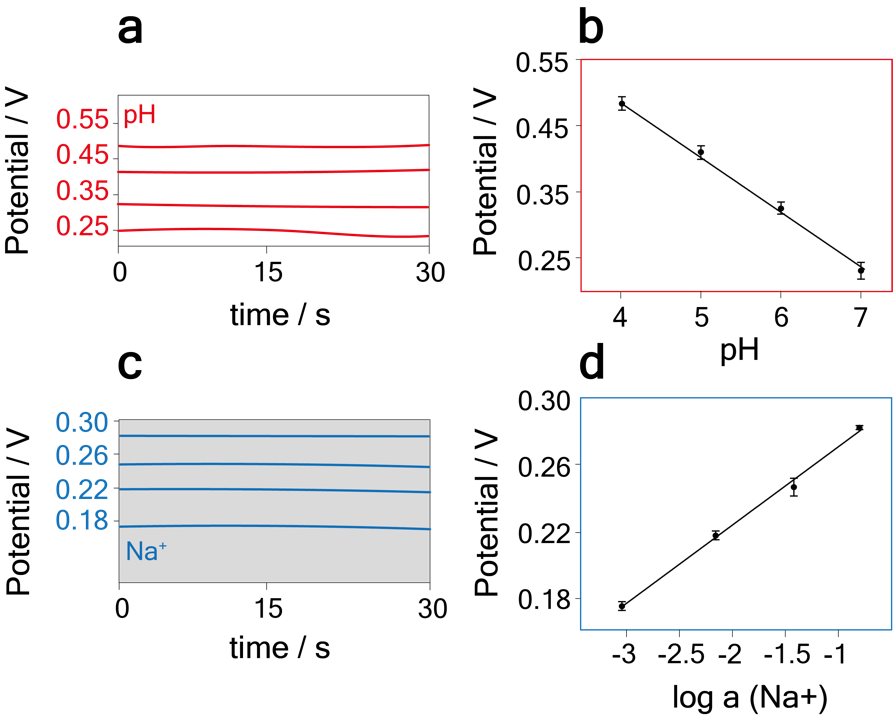
**

**Table S2.** Wearable sensors for the detection of various analytes in sweat during physical activity

| ***Sensor*** | ***Detection technique*** | ***Analytes*** | ***Microfluidics substrate*** | ***Microfluidic fabrication*** | ***Microfluidic***  ***architecture*** | ***Linear range / sensitivity in standard solution*** | ***Real sample measurement time*** | ***Ref*** |
| --- | --- | --- | --- | --- | --- | --- | --- | --- |
|  |  |  |  |  |  |  |  |  |
| SPE | Amperometry | Lactate and glucose | PDMS | Photolithography for mold creation to pattern the ﻿to pattern the PDMS | 5 inlet, 1 reaction chamber | Lactate: 4-20 mM / ﻿29.6 μM/μA  Glucose: 2-10 mM / ---  (Both in artificial sweat) | On body test up to 26.6 minutes | *1* |
|  |  |  |  |  |  |  |  |  |
| PL-electrodes | Potentiometry (Na^+^) and admittance (sweat rate) | ﻿ Optimized for Na^+^ and sweat rate | ﻿ PDMS | Photolithography for mold creation to pattern the spiral shaped PDMS | Spiral configuration | Na^+^ : 15-60 mM / 56 mV/dec | On body test up to 46,67 minutes | 2 |
|  |  |  |  |  |  |  |  |  |
| SPE | Amperometry (Cortisol)  Potentiometry (Mg^2+^ and pH ) | ﻿ Cortisol, Mg^2+^,  and pH | PDMS | Photolithography for mold creation to pattern the ﻿to pattern the PDMS | 1 inlet, 1 reaction chamber and 5 small outlets | Cortisol: 1 nM-10 µM / 2751 nA/dec  Mg^2+^: 1-10^4^ µM / 47.3 mV/dec  pH: 3-7.4 / ---  (analytical features assessed without microfluidics) | On body test up to 360 minutes | 3 |
|  |  |  |  |  |  |  |  |  |
| ﻿ LEG-electrodes | DPV | Optimized for Tyr, Trp, BCAA | ﻿  PI sandwiched between double-sided and single-sided medical adhesives | CO_2_ laser patterning and cutting | Multiple layers, multiple inlets into a reaction chamber to collect iontophoresis produced sweat. One outlet from the reaction zone | Tyr: 0-400 µM / 0.63 µA µM^−1^ cm^−2^  Trp: 0-400 µM / ﻿0.71 µA µM^−1^ cm^−2^  BCAA: 10-1000 µM /  (analytical features assessed without microfluidics) | On body test up to 60 or 100 minutes depending on the sport activity | 4 |
|  |  |  |  |  |  |  |  |  |
| LIG-electrodes | Potentiometry | Na^+^, K^+^ | ﻿  PET sandwiched between double-sided and single-sided medical  adhesives | CO_2_ laser patterning and cutting | Multiple layers, one inlet into a sensing chamber and one outlet | Na^+^: 5-160 mM / 67.43 mV/dec  K^+^: 0.625-40 mM / 30.42 mV/dec | On body test up to 33,33 minutes | 5 |
|  |  |  |  |  |  |  |  |  |
| SPE | Potentiometry | Na^+^, K^+^ | Cotton threads extracted from medical gauze and absorbent pad | Encapsulation in plastic holder | ﻿Sorbent material as sweat sampler | K^+^: 10^-4^-10^-1^ M / ﻿57.61 mV/dec  Na^+^: 10^-4^-10^-1^ M / 61.13 ﻿﻿mV/decade | On body test up to 90 minutes | 6 |
|  |  |  |  |  |  |  |  |  |
| SPE | Potentiometry | Na^+^, K^+^ | Whatman #4 Filter Paper | Wax printing | Origami multilayered configuration  for sweat collection, vertical diffusion, and evaporation | K^+^: 1-32 mM / ﻿58.68 mV  Na^+^: 8-128 mM / ﻿62.12 mV  (analytical features assessed without microfluidics) | On body test up to 30 minutes c.a. | 7 |
|  |  |  |  |  |  |  |  |  |
| SPE | DPV | Glucose, Lactate | ﻿Whatman chromatography paper #1 | Wax printing | Origami multilayered configuration for vertical diffusion, and evaporation | ﻿﻿Lactate: 0.3-20.3 µM / ﻿0.49 μA/mM  Glucose: and 0.08-1.25 mM / ﻿2.4 nA/μM | On body test up to 30 minutes | 8 |
|  |  |  |  |  |  |  |  |  |
| SPE | Potentiometry | Na^+^, pH | Scottex® tissue paper | CO_2_ laser cutting | Butterfly-shape consisting of sampling and waste zone | Na^+^: 10^-4^-10^-1^ M / 56 ± 5 mV/decade  pH: 4-7 / - (80 ± 4) mV/pH | On body test up to 40 minutes | This work |

| ﻿BCAA: Elevated branched-chain amino acids, DPV: differential pulse voltammetry, LEG: Laser-engraved graphene, LIG: Laser-induced graphene, PET: Polyethylene terephthalate, PDMS: polydimethylsiloxane, PI: polyimide, PL: Photolithography-fabricated, SPE: Screen-printed electrode. |
| --- |

1) A. Martín, J. Kim, J.F. Kurniawan, J.R. Sempionatto, J.R. Moreto, G. Tang, A.S. Campbell, A. Shin, M.Y. Lee, X. Liu, J. Wang, Epidermal Microfluidic Electrochemical Detection System: Enhanced Sweat Sampling and Metabolite Detection, ACS Sens. 2 (2017) 1860–1868. https://doi.org/10.1021/acssensors.7b00729.

2) H.Y.Y. Nyein, L.-C. Tai, Q.P. Ngo, M. Chao, G.B. Zhang, W. Gao, M. Bariya, J. Bullock, H. Kim, H.M. Fahad, A. Javey, A Wearable Microfluidic Sensing Patch for Dynamic Sweat Secretion Analysis, ACS Sens. 3 (2018) 944–952. https://doi.org/10.1021/acssensors.7b00961.

3) H. Zhao, X. Zhang, Y. Qin, Y. Xia, X. Xu, X. Sun, D. Yu, S.M. Mugo, D. Wang, Q. Zhang, An Integrated Wearable Sweat Sensing Patch for Passive Continuous Analysis of Stress Biomarkers at Rest, Adv. Funct. Materials. 33 (2023) 2212083. https://doi.org/10.1002/adfm.202212083.

4) M. Wang, Y. Yang, J. Min, Y. Song, J. Tu, D. Mukasa, C. Ye, C. Xu, N. Heflin, J.S. McCune, T.K. Hsiai, Z. Li, W. Gao, A wearable electrochemical biosensor for the monitoring of metabolites and nutrients, Nat. Biomed. Eng 6 (2022) 1225–1235. https://doi.org/10.1038/s41551-022-00916-z.

5) Y. Gai, E. Wang, M. Liu, L. Xie, Y. Bai, Y. Yang, J. Xue, X. Qu, Y. Xi, L. Li, D. Luo, Z. Li, A Self‐Powered Wearable Sensor for Continuous Wireless Sweat Monitoring, Small Methods 6 (2022) 2200653. https://doi.org/10.1002/smtd.202200653.

6) P. Pirovano, M. Dorrian, A. Shinde, A. Donohoe, A.J. Brady, N.M. Moyna, G. Wallace, D. Diamond, M. McCaul, A wearable sensor for the detection of sodium and potassium in human sweat during exercise, Talanta 219 (2020) 121145. https://doi.org/10.1016/j.talanta.2020.121145.

7) Q. Cao, B. Liang, X. Mao, J. Wei, T. Tu, L. Fang, X. Ye, A Smartwatch Integrated with a Paper‐based Microfluidic Patch for Sweat Electrolytes Monitoring, Electroanalysis 33 (2021) 643–651. https://doi.org/10.1002/elan.202060025.

8) M. Li, L. Wang, R. Liu, J. Li, Q. Zhang, G. Shi, Y. Li, C. Hou, H. Wang, A highly integrated sensing paper for wearable electrochemical sweat analysis, Biosensors and Bioelectronics 174 (2021) 112828. https://doi.org/10.1016/j.bios.2020.112828.
